# Morphological characteristics of diffuse idiopathic skeletal hyperostosis in the cervical spine

**DOI:** 10.1371/journal.pone.0188414

**Published:** 2017-11-20

**Authors:** Jessica T. Bakker, Jonneke S. Kuperus, Hugo J. Kuijf, F. Cumhur Oner, Pim A. de Jong, Jorrit-Jan Verlaan

**Affiliations:** 1 Department of Orthopaedics, University Medical Center Utrecht, Utrecht, The Netherlands; 2 Image Sciences Institute, University Medical Center Utrecht, Utrecht, The Netherlands; 3 Department of Radiology, University Medical Center Utrecht, Utrecht, The Netherlands; Medical College of Wisconsin, UNITED STATES

## Abstract

**Objectives:**

Diffuse idiopathic skeletal hyperostosis (DISH) is characterized by anterior ossification of the spine and can lead to dysphagia and airway obstruction. The morphology of the newly formed bone in the cervical spine is different compared to the thoracic spine, possibly due to dissimilarities in local vascular anatomy. In this study the spatial relationship of the new bone with the arterial system, trachea and esophagus was analyzed and compared between subjects with and without DISH.

**Methods:**

Cervical computed tomography (CT) scans were obtained from five patients with dysphagia and DISH and ten control subjects. The location of the vertebral and carotid arteries, surface area of the hyperostosis and distance between the vertebral body and the trachea and esophagus was assessed in the axial view.

**Results:**

The surface area of the newly formed bone was located symmetrically anterior to the vertebral body. The ossifications were non-flowing in the sagittal view and no segmental vessels were observed. Substantial displacement of the trachea/esophagus was present in the group with DISH compared to the controls.

**Conclusions:**

The hyperostosis at the cervical level was symmetrically distributed anterior to the vertebral bodies without a flowing pattern, in contrast to the asymmetrical flowing pattern typically found in the thoracic spine. The hypothesis that the vascular system acts as a natural barrier against new bone formation in DISH could be further supported with these findings. The significant ventral displacement of the trachea and esophagus may explain the mechanism of dysphagia and airway obstruction in DISH.

## Introduction

**Fig 1:**
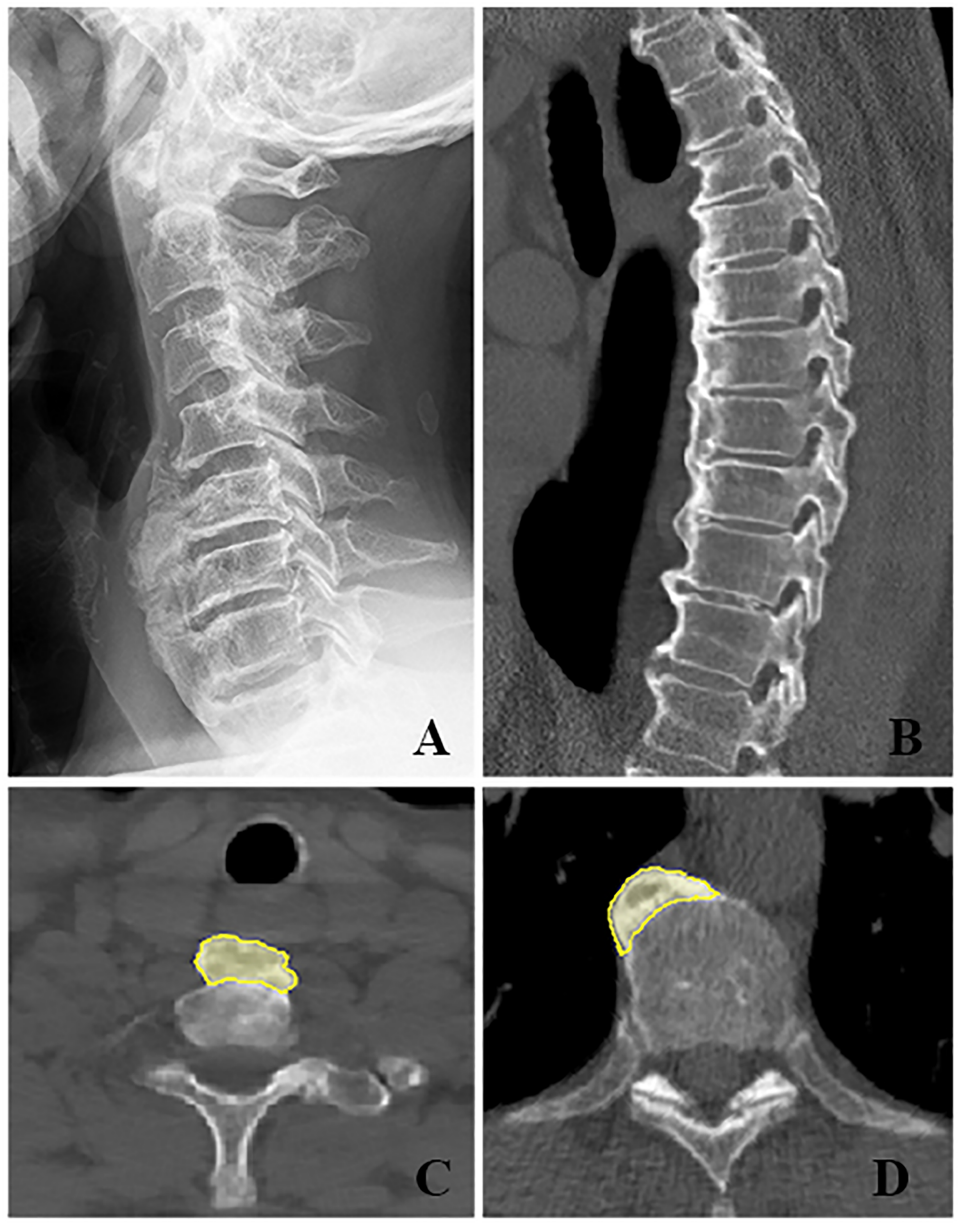
Typical examples of newly formed bone due to DISH in the cervical and thoracic spine. (A) Plain lateral radiograph shows a 69 year old male with DISH in the cervical spine. A solid formation of new bone is extending over at least four vertebral bodies. (B) Computed tomography (CT) visualizes the thoracic spine of a 72 year old male in the sagittal view. The scan shows a flowing ossification of the anterolateral spine with bridging over more than four contiguous vertebral bodies. The intervertebral discs and apophyseal joints are relatively intact in both images. (C + D) The CT scans in axial view demonstrate the differences in position of the new bone formation depending on the region. (C) The CT scan of the cervical spine corresponds to the radiographic image (A) and demonstrates symmetrical hyperostosis (yellow) anterior to the vertebral body and possible displacement of the trachea. (D) The axial CT of the mid thoracic spine in a 58 year old male with DISH shows the newly formed bone on the right anterolateral side with the aorta clearly located on the left anterolateral side.

Diffuse idiopathic skeletal hyperostosis (DISH) is a systemic condition characterized by ossification of the spine and peripheral entheses [1]. The typical changes related to DISH occur most frequently in the anterolateral part of the thoracic spine, contralateral to the descending thoracic aorta, with bridging ossifications that resemble flowing candle wax in the lateral view (Fig 1) [2,3].

The most commonly used diagnostic criteria for DISH, introduced by Resnick and Niwayama, are met if the ossifications span at least four contiguous vertebrae and do not involve gross degenerative changes in the intervertebral discs or apophyseal and sacroiliac joints [3]. Bone deposition in DISH in the cervical spine is frequently asymptomatic, but can result in dysphagia, aspiration, regurgitation, sleep apnea, upper respiratory tract infection, airway obstruction and difficult intubation [1,4,5]. Verlaan *et al*. noted in a systematic review that DISH of the cervical spine is an increasing and underappreciated phenomenon [4]. In a Dutch outpatient population older than 50 years the prevalence of DISH based on chest radiography was 22.7% in males and 12.1% in females [6]. The prevalence is expected to rise since DISH is correlated with older age, cardiovascular disease and underlying metabolic derangement [6,7].

It has been hypothesized that vascular structures act as a natural barrier for the formation of new bone in DISH [8]. This theory is supported by the development of thoracic ossifications mainly contralateral to the aorta. Moreover, the thoracic ossifications are thinnest where the segmental arteries run horizontally across the vertebral bodies, resulting in the typical undulating, flowing pattern [8]. In the cervical spine, however, the vertebral arteries and common, internal and external carotid arteries are located symmetrically and laterally to the vertebral bodies and segmental arteries (at least those of any significant caliber) crossing the vertebral bodies horizontally are absent [9]. If the hypothesis that large vessels prevent the formation of new bone in DISH is true, the newly formed bone in the cervical spine can be expected to be located in the midline and grow in a ventral direction, eventually leading to displacement of esophagus and trachea (Fig 1).

In this study the location of the newly formed bone in the cervical spine was assessed using computed tomography (CT) scans of subjects with symptomatic DISH and the spatial relationship of the new bone with the arterial system, trachea and esophagus was evaluated and compared to subjects without DISH.

## Materials and methods

### Subjects

This study was approved by the medical ethical review board of the University Medical Center Utrecht and the need for informed consent was waived (reference number 16/783). This study was performed using the strengthening the reporting of observational studies in epidemiology (STROBE) guidelines [10]. Computed tomography scans of five patients (one female, four males, 65–79 years of age) were selected retrospectively from our outpatient department database who had visited our department between 2010 and 2014. All patients had had complaints of dysphagia while eating solid food and were diagnosed with DISH according to the Resnick criteria [3]. Since only the cervical spine was included in the CT scans, evaluation of the sacroiliac joint was not possible although no signs pointing to the presence of ankylosing spondylitis or spondylosis, such as fused facet joints, were present in any of the patients. Ten subjects (4 females, 6 males, 57–90 years of age) were selected as control group to evaluate the normal anatomical location of the vascular system and trachea and esophagus. The control subjects had presented previously to the emergency department with stroke-like symptoms, however no pathologic findings (vascular or musculoskeletal) were detected on computed tomography angiography (CTA) of the brain and cervical region. All scans were anonymized prior to analysis.

### Imaging

The CT imaging of the five subjects with DISH was performed in supine position on a 64-slice calibrated CT scanner (120 kV, 300–375 mAs, 5 mm reconstructed slice thickness; Philips Medical Systems, Cleveland, OH). The study protocol of the CTA scans of the control group has been published previously [11].

Images were analyzed with custom made software based on MeVisLab 2.6 (MeVis Medical Solutions AG, Bremen, Germany [12]) [13]. The vertebral and carotid arteries were identified using Picture Archiving and Communication System (PACS) software (version 17.3, Sectra IDS7, Linköping, Sweden).

### Measurements

**Fig 2:**
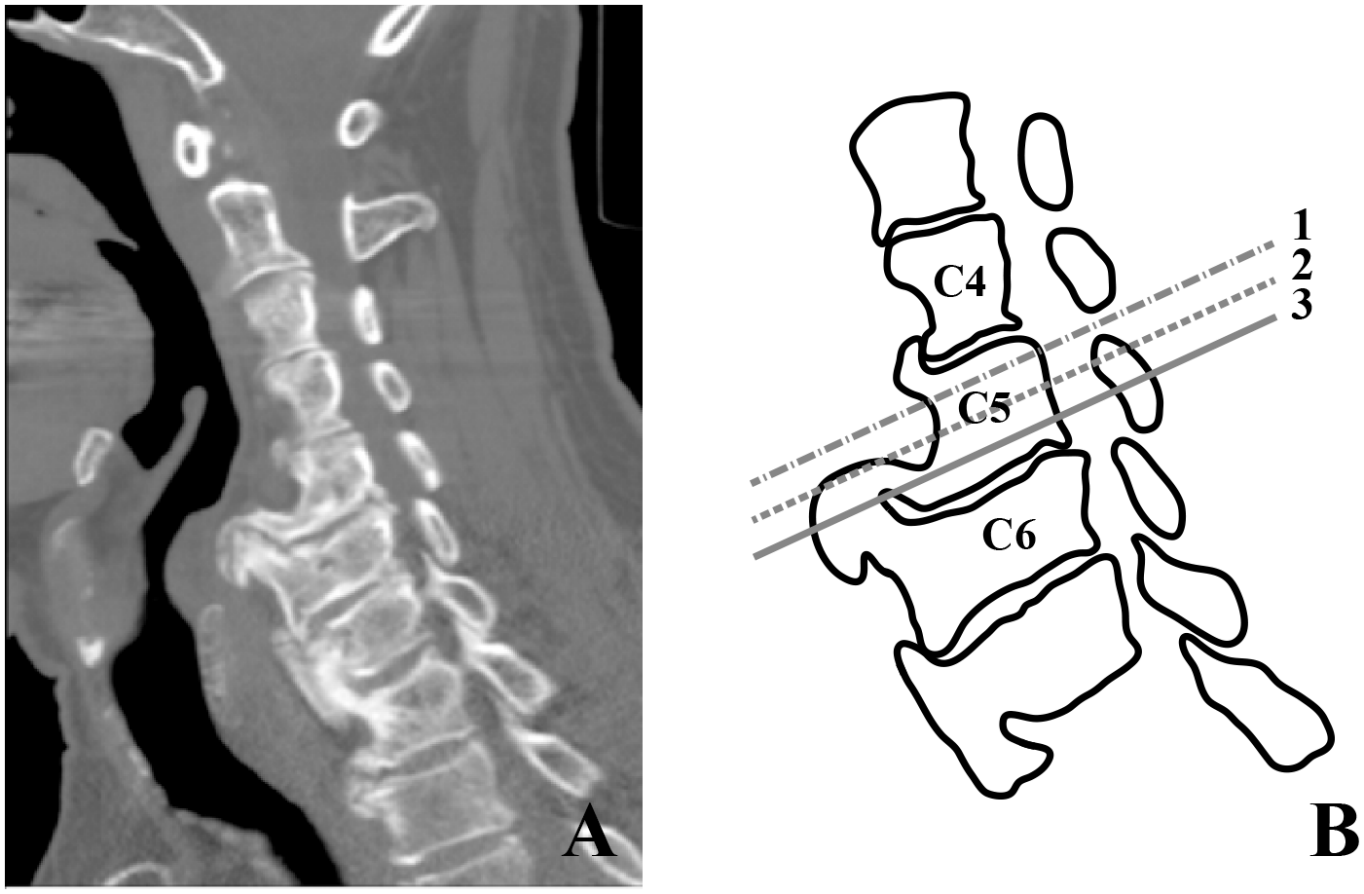
Graphical illustration of the planes used for the measurements. Measurements were performed at three levels in the C4, C5 and C6 vertebral bodies. The axial CT images were reconstructed to planes parallel to the endplate. (A) Sagittal CT image from a 69 year old male. The illustration (B) shows the three levels (C4, C5, C6) and three transverse locations at C5 (1, 2, 3) that were used for the measurements in the axial plane. The dashed line 1 shows the level adjacent to the cranial endplate, line 2 the mid-vertebral level and line 3 the level adjacent to the caudal endplate. The same approach (using the three lines for the transversal levels) was also used for the C4 and C6 vertebral body.

All measurements were performed by two independent observers. In the axial view, the images of all subjects were assessed to locate the position of the main arterial vessels, newly formed bone (if present) and trachea and esophagus. Vertebral levels C4, C5 and C6 were included because the hyperostosis is typically most extensive at these levels [4]. Three axial images, parallel to the endplates of the pertaining vertebral body, were selected: one adjacent to the cranial endplate, one at the mid-vertebral level and one adjacent to the caudal endplate (Fig 2).

**Fig 3:**
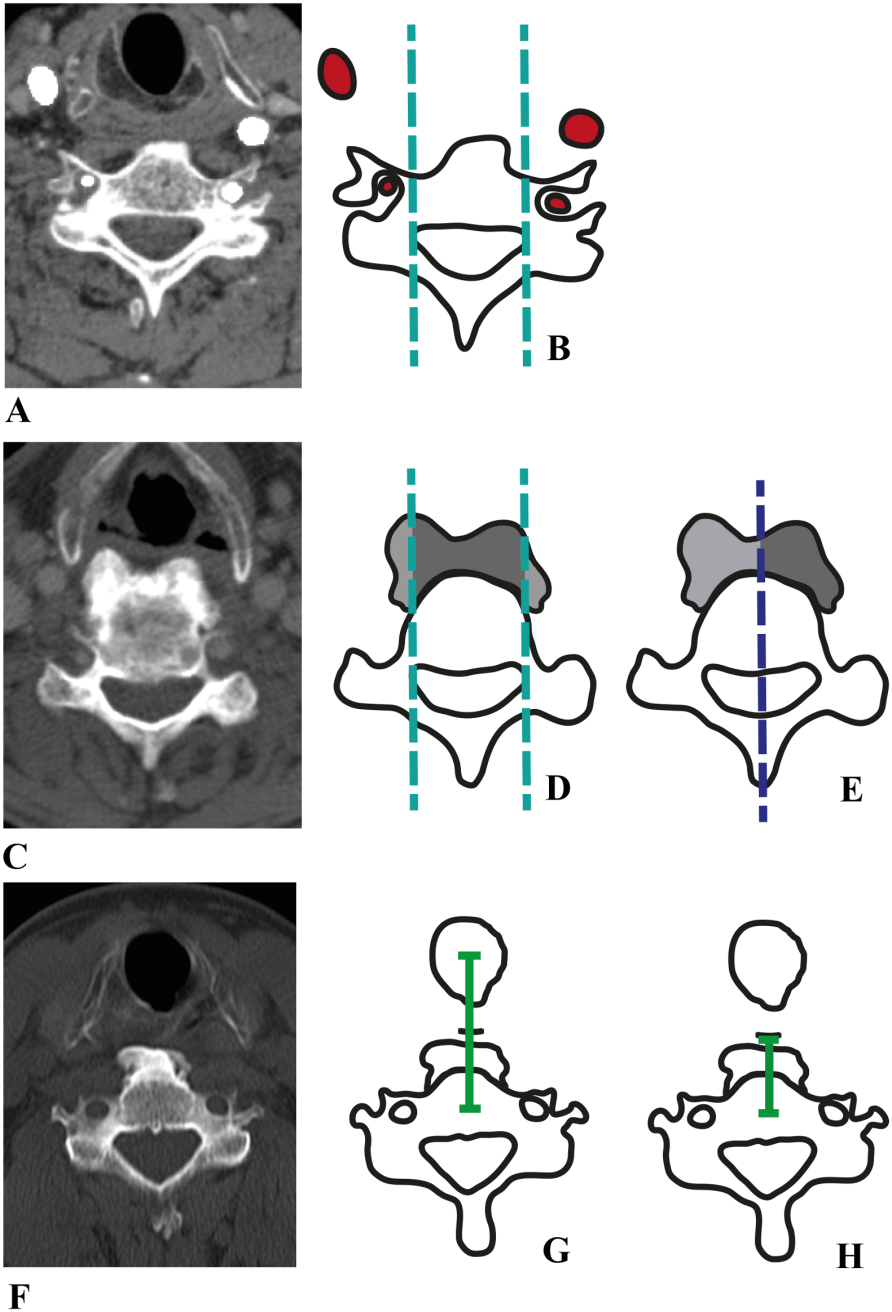
Graphical illustration of the measurements on the CT images. In (A) the CT scan is shown of a control subject with corresponding illustration (B). The parallel lateral lines are presented in light blue and carotid and vertebral arteries in red. CT scan (C) represents a male subject with DISH (72 years old) and matches illustrations (D and E). The parallel lateral lines (light blue) and the midsagittal anteroposterior (MAP) line (dark blue) were used to compare the different surface areas of newly formed bone (light/dark grey). CT scan (F) shows a male subject with DISH (61 years old) and corresponds to illustrations (G and H). The green lines demonstrate the distances between the center of the vertebral body and the trachea and esophagus, respectively.

In total, nine axial slices were available per subject to analyze the spatial relationship between arterial system, newly formed bone and esophagus/trachea. To describe the location of the different anatomical structures three lines were drawn on each axial image (Fig 3).

First, the midsagittal anteroposterior (MAP) line was defined as the line through the spinous process, center of the spinal canal and center of the vertebral body (Fig 3E). Additionally, two lines were drawn parallel to the MAP line intercepting the lateral borders of the spinal canal (Fig 3B and 3D). Bone formed between these two lateral lines was considered ‘anterior to the vertebral body’ and bone formed lateral from these two lines was considered ‘lateral to the vertebral body’.

The vertebral and carotid arteries were marked manually on the nine axial images of all subjects. The arterial blood vessels were considered to be located anterior and/or lateral to the vertebral body relative to the two parallel lateral lines (Fig 3B). The presence of segmental vessels (if any) was examined in the sagittal view. In the axial images of the subjects with DISH the outline of the area of newly formed bone was traced manually by the two observers using our software. The total surface area was calculated automatically. The two parallel lateral lines divided the total new bone area in an area anterior to the vertebral body and an area lateral to the vertebral body to assess the distribution of the new bone (Fig 3D). The left and right surface areas of the hyperostosis were compared relative to the MAP line to appraise the symmetry of new bone (Fig 3E).

Displacement of the trachea and esophagus was assessed by measuring the distance between the center of the vertebral body and the center of the trachea/esophagus in all subjects (Fig 3F and 3H). This measurement was performed in the transverse plane adjacent to the caudal endplate of C5.

### Statistics

Data were analyzed by SPSS version 20 software (IBM, Chicago, Illinois, USA). The mean surface area was calculated from the surface areas of the newly formed bone in the three axial images in the matching vertebral body and further statistical analyses were performed per vertebral level. The data was checked for normality using histograms, Q-Q plots and the Shapiro-Wilk test. The paired samples *t*-test or the Wilcoxon signed rank test was used to compare new bone anterior and lateral of the parallel lateral lines and left/right from the MAP line. The independent samples *t*-test was performed to assess differences in the distance of vertebral body to trachea/esophagus comparing subjects with and without DISH. The statistical significance level was set at p = 0.05 for all analyses and 95% confidence intervals (CI) were presented if relevant.

## Results

### Location of arterial vessels

**Fig 4:**
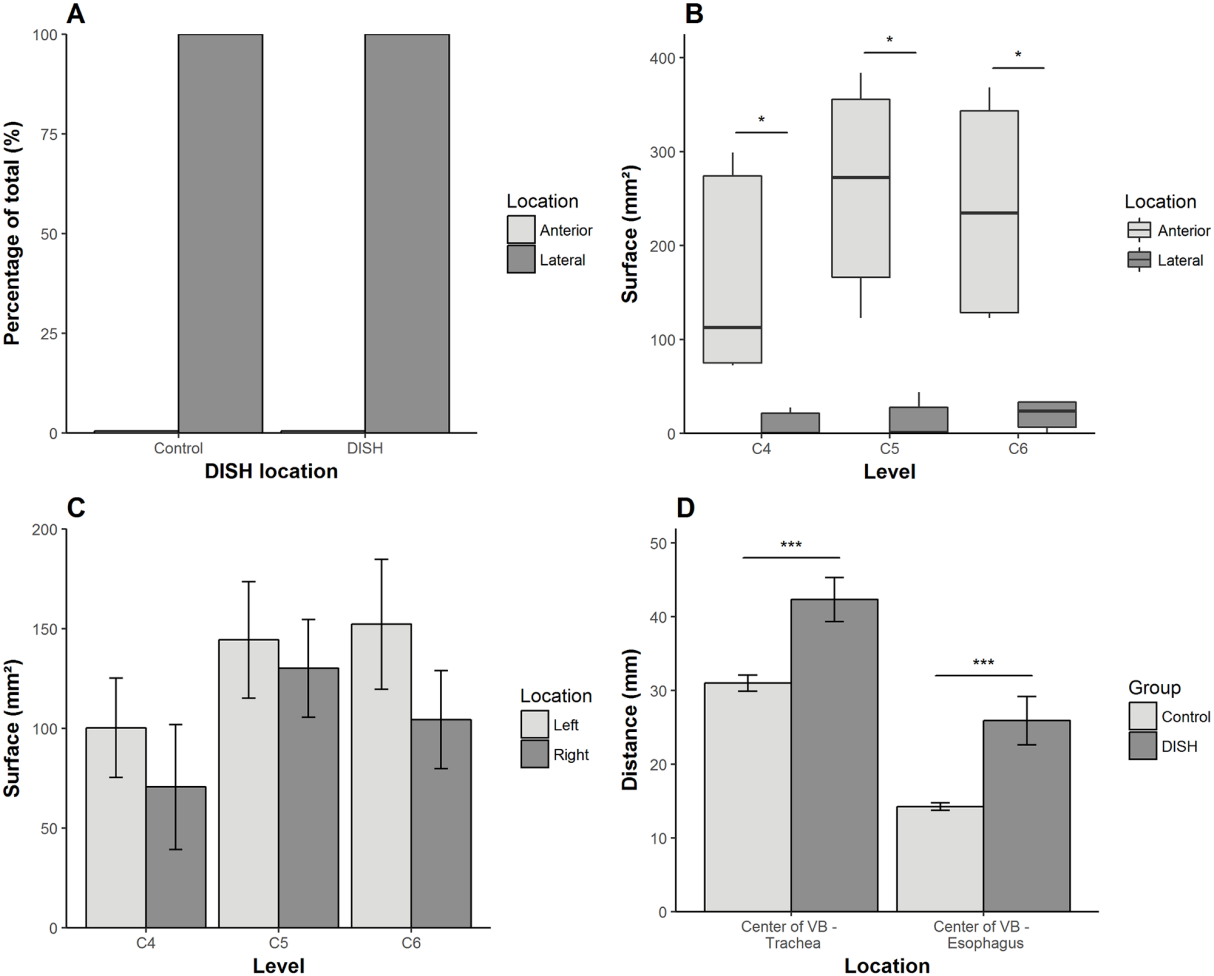
Results of the four different types of measurements. The location of the major arteries in the cervical spine was in all cases and at all levels lateral to the parallel lateral lines for the DISH and control group as shown in (A). The median total surface area of the newly formed bone per cervical level was significantly larger at the anterior location compared to the lateral location (B). There was no statistical difference between the left and right side of the MAP line (C). The distance between the center of the vertebral body and the trachea/esophagus was significantly larger in the group with DISH compared to the control group (D). The asterisk represents a p-value ≤ 0.05 and the triple asterisk represents a p-value ≤ 0.001. The error bars represent the standard error. VB–Vertebral body.

At every level in all subjects, the vertebral and carotid arteries were located outside the two parallel lateral lines and thus lateral to the vertebral body in the cervical spine (Fig 4A). Segmental vessels were not observed in any sagittal plane in any subject.

### Distribution of newly formed bone

In the subjects with DISH significantly more new bone was formed anterior than lateral relative to the vertebral body at all three vertebral levels of the cervical spine (Fig 4B). At level C4 the median size of the ossification area between the two parallel lateral lines was 113.0 mm^2^ (IQR 75.3–274.3) and outside the lines 0.2 mm^2^ (IQR 0–21.4; p = 0.043). At level C5 the median size of the surface area was 272.3 mm^2^ (IQR 166.1–355.5) between the two parallel lateral lines and 0.8 mm^2^ (IQR 0–27.8; p = 0.043) outside these lines. At level C6 the median size of the surface area between the two lines was 234.5 mm^2^ (IQR 128.7–343.2) and 23.9 mm^2^ (IQR 6.6–33.6; p = 0.043) outside the parallel lateral lines.

The mean size of the newly formed bone area left and right to the MAP line did not statistically differ for all levels (C4: p = 0.240, C5: p = 0.395, C6: p = 0.083), implying a roughly symmetrical distribution of the hyperostosis in DISH of the cervical spine (Fig 4C).

### Displacement of the trachea/esophagus

The mean distance from the center of the trachea to the center of the vertebral body was 42.3 mm (±6.6) in subjects with DISH and 31.0 mm (±3.4) in control subjects (p = 0.001; 95%CI 6–17; Fig 4D). The center of the esophagus to the center of the vertebral body measured 25.9 mm (±7.4) in the patients with DISH, compared to 14.3 mm (±1.6) in the control subjects (p<0.001; 95%CI 7–17). The distance between the vertebral body and the trachea/esophagus in the group with DISH was significantly larger compared to the corresponding distance in the control group.

## Discussion

The results from this study demonstrate that newly formed bone in DISH of the cervical spine occurs mainly anterior to the vertebral bodies. This is in contrast to the pattern of bone deposition at thoracic levels, where new bone is typically formed anterolaterally. Furthermore, the hyperostosis was symmetrically distributed relative to the sagittal midline, again in contrast to the asymmetrical bone depositions in the thoracic spine. The main vessels in the neck were located lateral to the vertebral bodies and segmental arteries were not detected on the sagittal CT images. These results support the hypothesis that arteries may act as a natural barrier for newly formed bone in DISH. The lack of crossing segmental vessels in the cervical spine may permit linear bone growth creating a bar-like structure in contrast to the flowing pattern typical for DISH in the thoracic spine where segmental vessels are present. The unrestricted formation of new bone anteriorly could explain the displacement of the trachea and esophagus in a ventral direction.

Already in 1950 Forestier and Rotes-Querol, discoverers of the condition currently known as DISH, described that spinal ossifications were often grooved by arterial branching, suggesting that the presence of (pulsating) vessels acted as a mechanical barrier for soft tissue ossification [2]. Their findings were supported by several authors reporting that the anterolateral ossification mass of the thoracic spine was found mainly on the right side of the vertebral column, away from the pulsating aorta [8]. This theory was backed by several observations of left sided hyperostosis of the thoracic spine in patients with situs viscerum inversus, which could be explained by the presence of a right sided aorta [14]. Our findings are in accordance with these studies, as the arteries in the cervical area were located lateral to the vertebral body and the newly formed bone in DISH was located mainly medially and anteriorly to the vertebral body.

The displacement of the trachea and esophagus observed in this study was, without doubt, related to the anteriorly located new bone and could help explain the mechanism of dysphagia and airway obstruction sometimes observed in patients with advanced DISH of the cervical spine. The often (sub)acute onset of symptoms, however, implies that mechanical obstruction by (slow) bone growth might not be the only contributor in the pathogenesis of these symptoms [4]. Several authors described other mechanisms that could also contribute to dysphagia or airway obstruction such as inflammation of soft tissues surrounding the ossification, nerve entrapment, limited movement of the epiglottis/larynx and retention of food due to the bone mass [15–18]. In the current study only the contribution of the mechanical obstruction was investigated and only the vertebral levels C4, C5 and C6 were analyzed on the CT images. Difficulties with endotracheal intubation in patients with DISH have been described in multiple case reports in the last decades [5,19–23]. As the presence of cervical hyperostosis could be asymptomatic (or mildly symptomatic), anesthesiologists could encounter unexpected difficulties in intubating patients with markedly displaced tracheas due to DISH [5]. Awake (fiberoptic) intubation is suggested to be a successful resolution in patients with a history of difficult intubation due to DISH [20,22].

The small number of cases with DISH included in this study is the likely result from the mostly asymptomatic natural course of this condition. In the literature only 204 patients with dysphagia and/or airway obstruction in combination with DISH are described between 1980 to 2009 [4]. In none of the patients with DISH included in the present study airway obstruction was present and all received surgery to remove the newly formed bone. The current observation of the spatial relationship between new bone and major vessels in the cervical spine corroborates previous findings from DISH in the thoracic spine although direct causal evidence cannot, in any way, be concluded from the present work. The anterior location of the newly formed bone could also be due to other causes such as repeated stretching of the degenerated anterior longitudinal ligament leading to subsequent ossification [1,3]. Evidence to support this theory is, however, also lacking. Forestier described the anterior longitudinal ligament to be pushed away by the bony outgrowths, suggesting that the ligament is not directly involved in DISH [2]. Until further studies are performed on the origin of the newly formed bone in DISH, the tentative hypothesis that new bone growth is limited by proximity of the arterial system may hold.

In conclusion, the new bone in individuals with DISH in the cervical spine is located anterior to the vertebral body in a symmetrical order in contrast to the anterolateral formation of bone in the thoracic spine with DISH. The symmetric lateral location of the cervical arteries and the lack of segmental vessels could explain the formation of an anterior bar-like layer of bone in DISH of the cervical spine without the typical flowing pattern observed in the thoracic spine. Both the trachea and esophagus were ventrally displaced in patients with DISH and this displacement is suspected to play an important role in the development of dysphagia. However, the causal relation between the new bone formation in DISH and presence of dysphagia or airway obstruction should be investigated in a longitudinal prospective study design with inclusion of patients with DISH with and without symptoms.

## Supporting information

S1 Table New bone area surface measurements. (SAV)

S2 Table Distance measurements between vertebral body and trachea/esophagus. (SAV)
